# Lipopolysaccharide-induced inflammation attenuates taste progenitor cell proliferation and shortens the life span of taste bud cells

**DOI:** 10.1186/1471-2202-11-72

**Published:** 2010-06-10

**Authors:** Zachary J Cohn, Agnes Kim, Liquan Huang, Joseph Brand, Hong Wang

**Affiliations:** 1 Monell Chemical Senses Center, Philadelphia, PA 19104-3308, USA; 2 Department of Biochemistry, School of Dental Medicine, University of Pennsylvania, Philadelphia, PA 19104, USA

## Abstract

**Background:**

The mammalian taste bud, a complex collection of taste sensory cells, supporting cells, and immature basal cells, is the structural unit for detecting taste stimuli in the oral cavity. Even though the cells of the taste bud undergo constant turnover, the structural homeostasis of the bud is maintained by balancing cell proliferation and cell death. Compared with nongustatory lingual epithelial cells, taste cells express higher levels of several inflammatory receptors and signalling proteins. Whether inflammation, an underlying condition in some diseases associated with taste disorders, interferes with taste cell renewal and turnover is unknown. Here we report the effects of lipopolysaccharide (LPS)-induced inflammation on taste progenitor cell proliferation and taste bud cell turnover in mouse taste tissues.

**Results:**

Intraperitoneal injection of LPS rapidly induced expression of several inflammatory cytokines, including tumor necrosis factor (TNF)-α, interferon (IFN)-γ, and interleukin (IL)-6, in mouse circumvallate and foliate papillae. TNF-α and IFN-γ immunoreactivities were preferentially localized to subsets of cells in taste buds. LPS-induced inflammation significantly reduced the number of 5-bromo-2'-deoxyuridine (BrdU)-labeled newborn taste bud cells 1-3 days after LPS injection, suggesting an inhibition of taste bud cell renewal. BrdU pulse-chase experiments showed that BrdU-labeled taste cells had a shorter average life span in LPS-treated mice than in controls. To investigate whether LPS inhibits taste cell renewal by suppressing taste progenitor cell proliferation, we studied the expression of Ki67, a cell proliferation marker. Quantitative real-time RT-PCR revealed that LPS markedly reduced Ki67 mRNA levels in circumvallate and foliate epithelia. Immunofluorescent staining using anti-Ki67 antibodies showed that LPS decreased the number of Ki67-positive cells in the basal regions surrounding circumvallate taste buds, the niche for taste progenitor cells. PCR array experiments showed that the expression of cyclin B2 and E2F1, two key cell cycle regulators, was markedly downregulated by LPS in the circumvallate and foliate epithelia.

**Conclusions:**

Our results show that LPS-induced inflammation inhibits taste progenitor cell proliferation and interferes with taste cell renewal. LPS accelerates cell turnover and modestly shortens the average life span of taste cells. These effects of inflammation may contribute to the development of taste disorders associated with infections.

## Background

Taste disorders, including taste loss and taste distortion, can substantially decrease quality of life and contribute to depression, anorexia and malnutrition [1-5]. While etiological studies have suggested the association of a range of conditions and diseases with taste impairment, the underlying cellular and molecular mechanisms of taste disorders remain largely unknown [2,5-8].

Several lines of evidence suggest that inflammation contributes to the development of taste disorders. First, inflammation, an immune response to infection, tissue damage, autoimmunity, and stress, is a common condition in a number of diseases associated with taste disorders, including upper respiratory infections, oral cavity infections, human immunodeficiency virus infection, autoimmune systemic lupus disease, Sjögren's disease, and cancer [3,9-14]. Second, multiple inflammation-related molecules, such as the Toll-like receptors (TLRs), the interferon (IFN) receptors, several chemokines, and cytokines, are expressed at higher levels in taste bud cells than in nontaste lingual epithelial cells [15-17]. Third, some inflammatory cytokines, such as IFNs, can alter gene expression and induce cell death in taste buds [15]. However, the effects of inflammation on taste bud structure and function have not been well characterized.

Mammalian taste buds, distributed on the surface of the oral cavity, harbour 50-100 cells, including taste receptor cells, supporting cells, and immature basal cells [18,19]. Taste receptor cells express the molecular machinery for detecting taste compounds and transmitting the signals, either directly and/or indirectly via other taste bud cells, to the peripheral gustatory nerves that innervate the taste buds [20,21]. During taste cell turnover, aged taste receptor cells degenerate and are replaced by new receptor cells differentiated from the basal cells. Although the average life span of taste cells is approximately 10 days [22-25], recent studies suggest that some taste cells can last more than 3 weeks in the buds [26,27]. Several cell death-related proteins, including the tumor suppressor protein p53, Bax (BCL2-associated X proteins), and caspases, are expressed in the taste buds [17,28,29]. However, it is unclear what mechanism initiates the cell death pathways and, therefore, determines the life span of taste cells.

To maintain structural stability and cell type equilibrium, taste progenitor cells give rise to newborn cells, which enter taste buds and differentiate into different types of mature taste bud cells. Little is known about the regulation of progenitor cell proliferation, immature cell differentiation, and taste cell degeneration. Some experimental manipulations can perturb these steps of taste bud turnover and disrupt the structural homeostasis. For instance, denervation of peripheral gustatory nerves induces extensive taste cell degeneration by apoptosis, which leads to the disappearance of taste buds [30-33]. On the other hand, dietary sodium restriction during pre- and postnatal development increases the latency for newborn cells to enter taste buds as well as taste cell life span and turnover periods [34]. The effect of inflammation on taste progenitor cell proliferation and taste bud cell turnover, however, has not yet been reported.

Inflammation mediated by TLR signalling promotes neurodegeneration and has been implicated in neurodegenerative diseases [35-38]. In addition, inflammatory stimuli (e.g., LPS) and proinflammatory cytokines, such as tumor necrosis factor (TNF)-α and interleukin (IL)-6, affect brain neurogenesis by modulating neural progenitor cell proliferation, newborn cell survival, and neural differentiation [39-43]. Taste sensory cells are epithelial cells with neuronal properties [44]. Taste progenitor cells and basal cells express genes such as *Sox2 *and *Mash1 *that are also involved in cell-fate determination and differentiation in the nervous system, suggesting that taste bud cells may share cell renewal mechanisms with neurons [45-47]. In this study, we investigated the effect of inflammation on taste progenitor cell proliferation and taste cell turnover using the LPS-induced acute inflammation model. Our results show that LPS strongly suppresses the expression of cyclin B2 and E2F1, two critical cell cycle regulators, in circumvallate and foliate epithelia. Accordingly, LPS markedly attenuates taste progenitor cell proliferation as shown by BrdU-labeling experiments and Ki67 immunostaining. Furthermore, LPS-induced inflammation curtails the average life span of taste bud cells.

## Results

### LPS stimulates the expression of inflammatory cytokines in taste tissues

**Figure 1:**
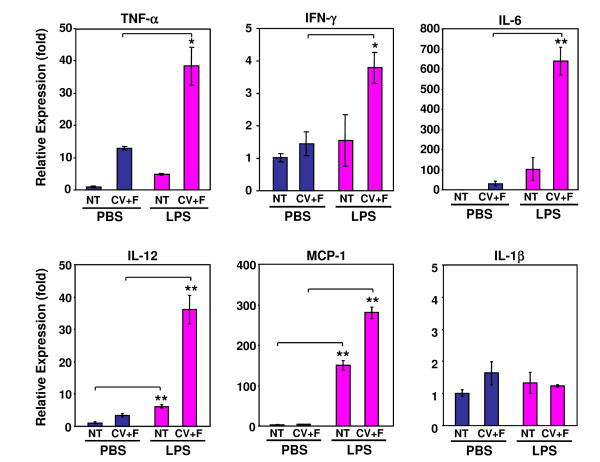
**Systemic inflammation induced by LPS stimulates the expression of inflammatory cytokines and the chemokine MCP-1 in circumvallate and foliate epithelia**. Quantitative real-time RT-PCR analysis of the expression of TNF-α, IFN-γ, IL-6, IL-12, MCP-1, and IL-1β in nontaste (NT) and circumvallate and foliate-containing (CV+F) lingual epithelia 6 h after intraperitoneal injection of PBS or LPS. Relative gene expression levels were shown (fold). Expression in nontaste epithelium from the PBS group was arbitrarily set to 1. β-actin was used as the endogenous control gene for relative quantification. Error bars represent SEM. * *p *< 0.05; ** *p *< 0.005.

Intraperitoneal injection of LPS, a Gram-negative bacterial cell wall component, induces systemic inflammation characterized by the production of a spectrum of cytokines [48-50]. To investigate whether LPS injection can induce an inflammatory response in taste tissues, we examined the expression of several inflammatory cytokines, including TNF-α, IFN-γ, IL-1β, IL-6, and IL-12, and the chemokine monocyte chemoattractant protein (MCP)-1, in circumvallate and foliate papillae. Quantitative real-time reverse transcription-polymerase chain reaction (RT-PCR) analysis showed that 6 h after intraperitoneal LPS injection, the expression levels of TNF-α, IFN-γ, IL-6, IL-12, and MCP-1 were all elevated in circumvallate and foliate epithelia (Figure 1; see Table 1 for qPCR primers and Additional File 1 for DNA gel images). The expression levels of IL-1β in LPS-treated samples were not significantly different from those in PBS-treated samples 6 h after LPS injection (Figure 1). It remains to be determined whether LPS stimulates the expression of IL-1β at other time points after treatment. These results suggest that systemic administration of LPS can induce a robust inflammatory response in taste epithelium, consistent with recent studies that demonstrate the preferential expression of various inflammatory receptors, signaling proteins, and cytokines in taste buds [15-17]. MCP-1 expression in taste papillae can also be upregulated by gustatory nerve injury [51].

**Table 1:** RT-PCR primers used for gene expression analysis

Gene Name	GenBank Accession #	Forward Primer	Reverse Primer	Product (bp)
**Cytokines**:				

IFN-γ	NM_008337	AGCAACAGCAAGGCGAAAA	CTGGACCTGTGGGTTGTTGA	71

IL-1β	NM_008361	GTAATGAAAGACGGCACACC	ATTAGAAACAGTCCAGCCCA	270

IL-6	NM_031168	TCATATCTTCAACCAAGAGGTA	CAGTGAGGAATGTCCACAAACTG	230

IL-12	NM_008352	CGAATCCAGCGCAAGAAAGA	GGAACACATGCCCACTTGCT	173

MCP-1	NM_011333	CAGCAGGTGTCCCAAAGAAG	GACCTTAGGGCAGATGCAGT	165

TNF-α	NM_013693	CCTCACACTCAGATCATCTTCTCA	TGGTTGTCTTTGAGATCCATGC	147

**Cell cycle markers and regulators**:				

Ki67	NM_001081117	TCTGATGTTAGGTGTTTGAG	CACTTTTCTGGTAACTTCTTG	177

Cyclin B2	NM_007630	CAACCGTACCAAGTTCATCG	CATACAGGATCTGAGAAGCG	169

E2F1	NM_007891	ACCATCACCTCCCTCCACAT	TGGTGACAGTTGGTCCTCTT	127

**Endogenous control**:				

β-Actin	NM_007393	GATTACTGCTCTGGCTCCTA	ATCGTACTCCTGCTTGCTGA	142

GenBank accession numbers for the sequences used to select the corresponding primers are given. The sizes of the predicted PCR products are indicated in base pairs.

**Figure 2:**
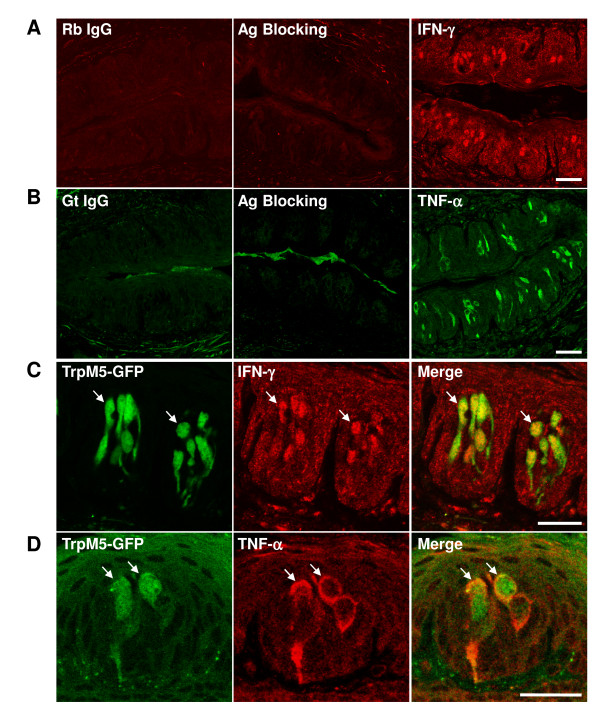
**TNF-α and IFN-γ immunoreactivity in the mouse circumvallate epithelium**. Immunofluorescent staining of mouse circumvallate papillae taken 6 h after LPS-injection. A, Confocal fluorescent images of circumvallate sections stained with control normal rabbit IgG (Rb IgG), affinity-purified rabbit antibody against IFN-γ (IFN-γ), or antibody against IFN-γ pre-incubated with purified recombinant murine IFN-γ (Ag Blocking). Cy3-conjugated anti-rabbit secondary antibody was used. B, Confocal fluorescent images of circumvallate sections stained with control normal goat IgG (Gt IgG), affinity-purified goat antibody against TNF-α (TNF-α), or antibody against TNF-α pre-incubated with purified recombinant murine TNF-α (Ag Blocking). Alexa 488-conjugated anti-goat secondary antibody was used. C, D, Colocalization of IFN-γ (C) or TNF-α (D) immunoreactivity (red) with TrpM5-GFP-positive (green) taste bud cells (arrows). Cy3-conjugated anti-rabbit and Cy5-conjugated anti-goat secondary antibodies were used for IFN-γ and TNF-α immunostaining, respectively. Scale bars, 25 μm.

To characterize the types of cells that produce these inflammatory molecules in taste papillae, we performed immunostaining using antibodies against TNF-α and IFN-γ. Both IFN-γ and TNF-α antibodies stained a subset of cells in the circumvallate taste buds after LPS treatment (Figure 2 A, B). Species-matched non-specific control antibodies did not show any specific staining in the taste buds. Pre-incubation of the antibodies with their corresponding antigens eliminated the staining of taste bud cells (Figure 2 A, B). Next, we performed colocalization studies using these antibodies on taste tissue sections from TrpM5-GFP transgenic mice (the expression of green fluorescent protein under the control of TrpM5 promoter) [52,53] (Figure 2 C, D). These experiments showed that the immunoreactivities of IFN-γ and TNF-α antibodies localized to TrpM5 expressing cells. These results suggest that some taste bud cells can produce inflammatory cytokines after LPS stimulation.

### LPS-induced inflammation inhibits taste bud cell renewal and shortens the lifespan of taste cells

**Figure 3:**
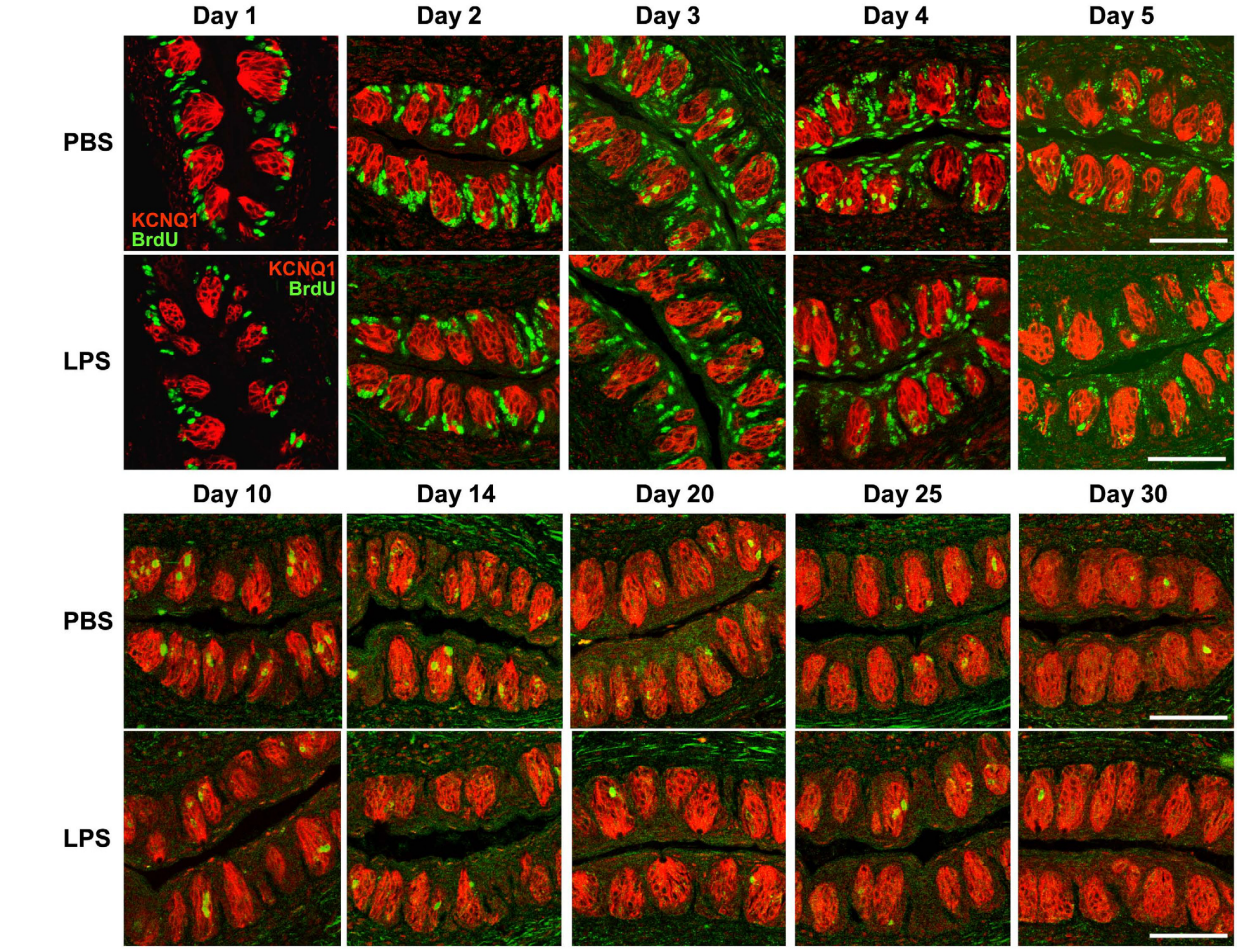
**Turnover of BrdU-labeled cells in circumvallate epithelium of PBS- or LPS-treated mice**. Circumvallate sections from mice injected with BrdU and either PBS or LPS were processed for immunofluorescent staining with antibodies against BrdU (green) and KCNQ1 (red). Representative confocal images (merged red and green channels) from both PBS and LPS groups are shown for selected time points. Scale bars, 75 μm.

To investigate the effects of inflammation on taste cell turnover, we used the well-established 5-bromo-2'-deoxyuridine (BrdU) pulse-chase method to follow the process of cell turnover [26,27]. In order to label an adequate number of taste cells, mice were injected with five doses of BrdU over a 12-h period. One dose of LPS or PBS (control) was injected intraperitoneally 1 h after the first BrdU injection. Taste tissues were collected at 1, 2, 3, 4, 5, 6, 7, 8, 10, 12, 14, 16, 18, 20, 25, and 30 days after the initial BrdU administration. Tissue sections containing circumvallate taste buds were processed for immunofluorescent staining using antibodies against BrdU as well as KCNQ1, a marker protein for taste bud cells [54]. Representative immunostaining images from both LPS- and PBS-treated groups are shown in Figure 3. Consistent with published studies [22,27], the majority of BrdU-positive cells reside in the basal layer of circumvallate epithelium outside of taste buds 1 day after BrdU injection for both LPS and PBS groups. From day 2 through day 5, most of the BrdU-positive cells in both groups gradually migrated from the basal to the apical regions of perigemmal epithelium, and only a small percentage of BrdU-positive cells were incorporated into the taste buds (Figure 3, days 1-5). Similar to previously published results [27], a small number of BrdU-positive taste cells were observed 25 and 30 days after BrdU injection (Figure 3), suggesting that some taste cells can survive much longer than the estimated average life span of taste bud cells (estimated to be 250 ± 50 hours) [22].

**Figure 4:**
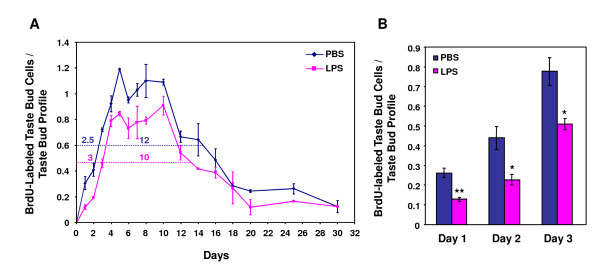
**LPS inhibits renewal and shortens average turnover period of taste bud cells**. A, Time course of the number of BrdU-labeled taste cells per circumvallate taste bud profile of PBS- or LPS-treated mice. Solid lines indicate the time course curves. Dashed lines show where the numbers of BrdU-labeled taste cells reached half-maximum values for each group. The left and right numbers above the dashed lines are the average times for cells to enter the taste buds (2.5 days for the PBS group and 3 days for the LPS group) and the average taste bud cell turnover periods (12 days for the PBS group and 10 days for the LPS group). LPS treatment slowed the entry of taste bud cells and shortened their turnover period. B, Entry of BrdU-labeled cells into taste buds on days 1-3 after BrdU injection. LPS treatment decreased the number of BrdU-labeled cells per taste bud profile compared with controls. Error bars indicate SEM. * *p *< 0.05; ** *p *< 0.005.

To quantitatively analyze the data, we counted the number of BrdU-labeled cells within the profiles of circumvallate taste buds. The counting criteria are described in detail in the Methods section. The average numbers of BrdU-positive cells per taste bud profile are summarized in Table 2, and the time course is plotted in Figure 4A. Based on the time-course curves, we estimated the average taste bud cell entry time, turnover period, and life span using previously described methods [23,34]. For the control group, our experiments showed that the average taste bud cell entry time was 2.5 days and the average taste cell turnover period was 12 days (Figure 4A). Both time periods were slightly longer than those reported by others [22,23], likely due to the differences in DNA labeling methods (^3^H-thymidine vs. BrdU) and injection schedules (one injection of ^3^H-thymidine vs. five injections of BrdU). Even given these differences, the traditional estimates and ours are remarkably similar.

**Table 2:** Counts of BrdU-labeled cells in circumvallate taste buds of PBS- and LPS-treated mice.

Days	PBS			LPS		
	
	No. of taste bud profiles examined	No. of BrdU-labeled taste bud cells	BrdU-labeled cells per taste bud profile	No. of taste bud profiles examined	No. of BrdU-labeled taste bud cells	BrdU-labeled cells per taste bud profile
1	291	89	0.31	287	34	0.12

2	373	153	0.41	435	83	0.19

3	430	310	0.72	356	164	0.46

4	328	303	0.92	357	282	0.79

5	352	420	1.19	341	289	0.85

6	344	328	0.95	371	272	0.73

7	306	316	1.03	303	237	0.78

8	305	337	1.10	320	254	0.79

10	346	378	1.09	371	338	0.91

12	337	225	0.67	365	198	0.54

14	368	237	0.64	329	137	0.42

16	331	161	0.49	356	138	0.39

18	320	91	0.28	359	97	0.27

20	409	100	0.24	403	48	0.12

25	342	90	0.26	345	57	0.17

30	331	41	0.12	358	44	0.12

Mice injected with BrdU and PBS or LPS were sacrificed at the indicated time points. Circumvallate sections were stained with antibodies against BrdU and KCNQ1. Confocal fluorescent images were used to count BrdU-labeled cells inside the taste bud profiles defined by the KCNQ1 staining. The total numbers of taste bud profiles examined and the BrdU-labeled taste bud cells counted, as well as the average number of BrdU-labeled cells per taste bud profile, are shown for each time point. These numbers were used to generate the time course curves shown in Figure 4A.

Compared with controls, LPS treatment altered several aspects of taste cell turnover: 1) the average taste bud cell entry time was slightly longer for the LPS group (3 days) than for the PBS group (2.5 days) (Figure 4A); 2) the number of BrdU-positive cells entering the taste buds was significantly reduced at multiple time points for the LPS group compared with the PBS group (Figure 4A, B); and 3) the turnover period for taste bud cells, an indication of the average time for a cell to stay in taste buds, was shorter for the LPS group (10 days) than for the PBS group (12 days) (Figure 4A). Together, these results suggest that LPS-induced inflammation inhibits taste bud cell renewal and accelerates taste cell turnover.

**Figure 5:**
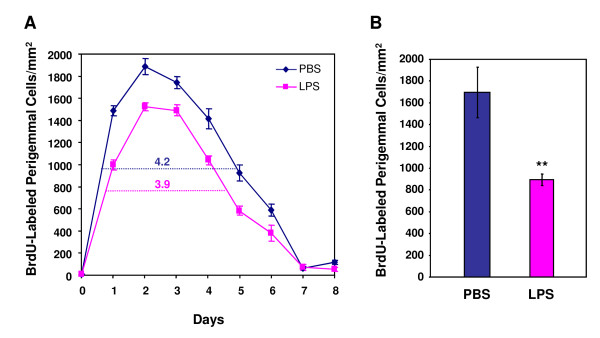
**The effects of LPS on perigemmal keratinocyte turnover**. A, Time course of the number of BrdU-labeled perigemmal cells/mm^2 ^in circumvallate epithelium of PBS- or LPS-treated mice. Solid lines indicate the time course curves. Dashed lines show where the numbers of BrdU-labeled perigemmal cells reached half-maximum values for each group. The numbers above the dashed lines are the average turnover periods for the perigemmal keratinocytes in the circumvallate epithelium (4.2 days for the PBS group and 3.9 days for the LPS group). B, Average number of BrdU-labeled cells/mm^2 ^in the perigemmal region of circumvallate epithelium 1 day after BrdU injection. LPS treatment significantly decreased the number of BrdU-labeled perigemmal cells. Error bars indicate SEM. ** *p *< 0.005.

We also counted BrdU-labeled cells in the perigemmal regions of circumvallate epithelium (Table 3, Figure 5). Consistent with published studies [27], the turnover periods of perigemmal keratinocytes were much shorter than those of taste bud cells, with a turnover period estimated to be 4.2 days for the PBS group and 3.9 days for the LPS group (Figure 5A). Similar to its effect on taste bud cells, LPS treatment also significantly decreased the number of BrdU-labeled cells in the perigemmal cell population (Figure 5A, B).

**Table 3:** Counts of BrdU-labeled perigemmal cells in circumvallate papillae of PBS- and LPS-treated mice.

Days	PBS			LPS		
	
	No. of BrdU-labeled perigemmal cells	**Total area (μm**^**2**^**)**	**BrdU-labeled perigemmal cells per mm**^**2**^	No. of BrdU-labeled perigemmal cells	**Total area (μm**^**2**^**)**	**BrdU-labeled perigemmal cells per mm**^**2**^
1	531	3.57 × 10^5^	1487.4	304	3.05 × 10^5^	998.4

2	655	3.47 × 10^5^	1890.3	673	4.41 × 10^5^	1526.1

3	732	4.2 × 10^5^	1742.9	438	2.94 × 10^5^	1489.8

4	505	3.57 × 10^5^	1414.6	436	4.2 × 10^5^	1038.1

5	427	4.62 × 10^5^	924.2	243	4.2 × 10^5^	578.6

6	233	3.99 × 10^5^	584.0	173	4.62 × 10^5^	374.5

7	26	4.62 × 10^5^	56.3	24	3.78 × 10^5^	63.5

8	37	3.36 × 10^5^	110.1	18	3.78 × 10^5^	47.6

Experiments were carried out as described in Table 2. BrdU and KCNQ1 immunostaining images were used to count BrdU-labeled perigemmal cells in the circumvallate epithelium of PBS- or LPS-treated mice. The total numbers of BrdU-labeled perigemmal cells and the total areas of circumvallate epithelium counted, as well as the average numbers of BrdU-labeled cells/mm^2 ^of circumvallate epithelium, are shown for each time point. These numbers were used to generate the time course curves shown in Figure 5A.

### LPS inhibits proliferation of progenitor cells for taste buds

Recent studies suggest that taste bud cells and surrounding keratinocytes are derived from a common progenitor cell population residing in the basal regions surrounding the taste buds [47,55]. This group of progenitor cells expresses cell proliferation markers as well as cell lineage regulators, such as Patched1, Sox2, Trp63, and Ki67 [27,47,56-58], reflecting their proliferative properties and roles in cell fate determination. Since LPS treatment inhibits the renewal of both taste bud cells and perigemmal keratinocytes (Figures 4, 5), it is possible that the common progenitor cells for both cell lineages are affected.

**Figure 6:**
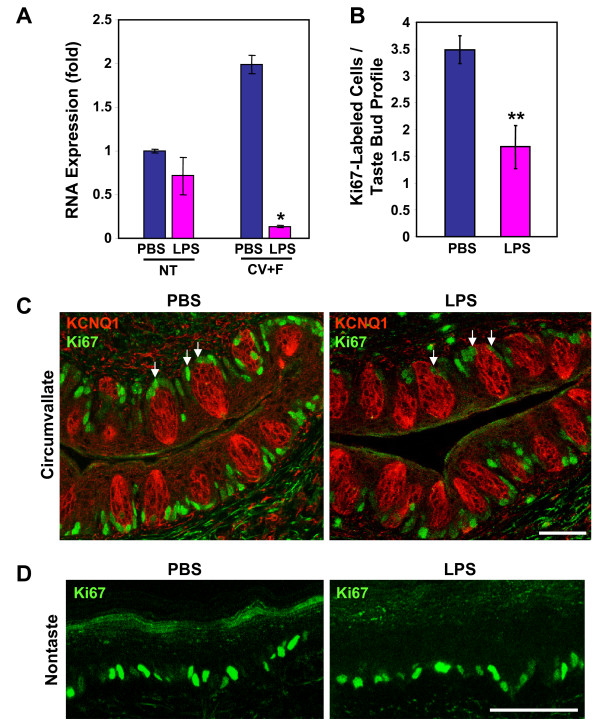
**LPS inhibits taste progenitor cell proliferation**. A, Quantitative real-time RT-PCR analysis of Ki67 expression in nontaste (NT) and circumvallate and foliate-containing (CV+F) lingual epithelia 24 h after intraperitoneal injection of PBS or LPS. β-actin was used as the endogenous control for relative quantification, and the expression level of Ki67 in nontaste epithelium of PBS-treated mice was arbitrarily set to 1. LPS strongly inhibited the expression of Ki67 in the circumvallate and foliate epithelia. B, The number of Ki67-labeled cells per taste bud profile in the circumvallate epithelium. Only the Ki67-labeled cells that were adjacent to a taste bud were counted (a few examples are indicated by arrows in C). LPS treatment significantly reduced the number of Ki67-labeled cells in the circumvallate epithelium. Error bars indicate SEM. * *p *< 0.05; ** *p *< 0.005. C, Confocal fluorescent images of circumvallate papillae stained with antibodies against KCNQ1 (red) and Ki67 (green). LPS treatment strongly reduced the staining intensity and the number of cells labeled with Ki67 antibody. D, Representative confocal images of Ki67 immunostaining of the nontaste lingual epithelium from PBS- or LPS-treated mice. Scale bars, 50 μm.

To investigate whether LPS suppresses proliferation of progenitor cells for taste buds, we examined the expression of Ki67, a cell proliferation marker expressed in all active stages of the cell cycle [59]. Quantitative real-time RT-PCR revealed that the expression level of Ki67 mRNA was dramatically reduced in the circumvallate and foliate epithelia 24 h after LPS treatment (Figure 6A). In contrast, Ki67 expression levels were not significantly different in nontaste lingual epithelium from PBS- versus LPS-treated mice. To investigate whether the decreased expression of Ki67 by LPS in taste epithelium was due to a reduction of proliferating taste progenitor cells, we performed immunostaining using antibodies against Ki67. We confirmed that in the circumvallate epithelium, the Ki67 antibody recognized a group of cells in the basal regions surrounding taste buds, the niche for taste progenitor cells (Figure 6C). Ki67-immunoreative cells were also positive for K14 immunostaining (see Additional File 2), a marker for taste progenitor cells [47]. LPS treatment markedly reduced the number of Ki67-positive cells and the intensity of Ki67 staining in the basal regions surrounding circumvallate taste buds (Figure 6B, C). In contrast, Ki67 staining was comparable in nontaste lingual epithelia from PBS- and LPS-treated mice (Figure 6D). Together, the results from these experiments and BrdU-labeling experiments demonstrate that LPS-induced inflammation inhibits taste progenitor cell proliferation.

### LPS suppresses the expression of cyclin B2 and E2F1 in taste epithelium

**Figure 7:**
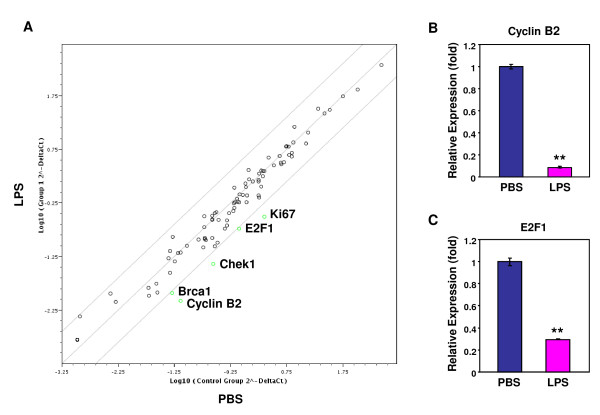
**LPS-induced inflammation downregulates the expression of cyclin B2 and E2F1 in the circumvallate epithelium**. A, Scatter plot summarizing gene expression analysis using the Mouse Cell Cycle RT^2 ^Profiler PCR Array. The expression of 84 genes involved in cell cycle regulation was analyzed by real-time RT-PCR in circumvallate and foliate epithelial samples prepared 24 h after PBS (x-axis) or LPS (y-axis) injection. Each circle represents a gene in the PCR array. The middle diagonal gray line indicates fold change of 1 (no change of gene expression between PBS and LPS groups). Circles above this gray line indicate increased gene expression, and circles below, decreased expression, in LPS samples versus PBS samples. The two outside gray lines indicate a fold change of 4. The five green circles represent genes that showed >4-fold decreased expression in LPS samples. B, C, Independent real-time RT-PCR expression analyses of cyclin B2 (B) and E2F1 (C) in circumvallate and foliate epithelia prepared 24 h after PBS or LPS injection. LPS significantly decreased the expression of both genes in circumvallate and foliate epithelia. Error bars indicate SEM. ** *p *< 0.005.

To further investigate the effects of LPS-induced inflammation on taste progenitor cell proliferation, we carried out quantitative real-time RT-PCR analysis using the Mouse Cell Cycle RT^2 ^Profiler PCR Array from SABiosciences. This PCR array contains primers for 84 genes involved in cell cycle regulation as well as several genes as endogenous controls (see Additional File 3: Mouse cell cycle PCR array data). Figure 7A compares the expression of these genes in the circumvallate and foliate epithelia from PBS- and LPS-treated mice. Only the genes that displayed >4-fold decrease in expression in LPS samples are labeled: Ki67 (7.20-fold), E2F1 (4.25-fold), Chek1 (checkpoint kinase 1 homolog) (6.88-fold), Brca1 (breast cancer associated gene 1) (4.48-fold), and cyclin B2 (8.78-fold). Additional File 3 lists all the genes examined by PCR array analysis.

The decreased expression of Ki67 by LPS is consistent with the result shown in Figure 6A, which was based on RT-PCR experiments using independently designed primers (see Table 1). Of the other four genes, Chek1 and Brca1 are involved in DNA damage response and repair [60], whereas Cyclin B2 and E2F1 directly regulate cell cycle progression. Cyclin B2, one of the three mammalian B-type cyclins, is an interacting partner for cyclin-dependent kinase 1 (Cdk1) [61]. Cdk1/cyclin B2 complexes are essential for the reorganization of the Golgi apparatus during mitosis[62]. E2F1 is a transcription factor that regulates cell cycle progression by activating the transcription of numerous genes required for cell division [63]. Decreased expression of cyclin B2 and E2F1 may contribute to the suppression of taste progenitor cell proliferation by LPS. To validate the data generated through the PCR array experiment, we carried out real-time RT-PCR reactions for cyclin B2 and E2F1 using independently designed primers (see Table 1). The results showed that the expression of these two critical cell cycle regulators was indeed inhibited by LPS (Figure 7B, C).

## Discussion

### Inflammation and taste progenitor cell proliferation

Inflammation is a complex process involving the interplay of numerous factors such as cytokines (both pro-inflammatory and anti-inflammatory) and chemokines. The effects of inflammation on cell proliferation are multifaceted, with different outcomes depending on cell types, disease models, and the inflammatory factors involved [43,64,65]. In adult brain hippocampus, LPS-induced inflammation is detrimental to neurogenesis, whereas deficiency of TLR4, the receptor for LPS, results in enhanced progenitor cell proliferation and neuronal differentiation [39,40,42]. However, inflammation induced by ischemia and mechanical injury can stimulate proliferation and promote neurogenesis [43]. Similarly, although IL-6 is shown to inhibit neural progenitor cell proliferation and newborn cell survival, TNF-α can either impair proliferation and survival or support neurogenesis, depending on the expression of TNF receptors and the models employed [41,43,65,66]. The mechanisms by which inflammation mediates these varying effects on cell proliferation and survival are largely unknown.

Our results showed that LPS-induced inflammation stimulated the expression of several inflammatory cytokines in taste papillae (Figure 1). LPS treatment inhibited proliferation of taste progenitor cells (Figure 6) and reduced the number of newborn cells entering taste buds (Figure 4). In line with the hypothesis that taste bud cells and perigemmal keratinocytes share a common progenitor cell pool [47], LPS treatment also reduced the number of newborn perigemmal cells in the circumvallate epithelium (Figure 5). It is likely that the decreased expression of cyclin B2 and E2F1 (Figure 7), two crucial regulators of cell cycle progression, is involved in this inhibition. Yet, it is unclear what molecular pathways lead to the suppression of cyclin B2, E2F1, and Ki67 in the taste epithelium upon LPS treatment. Previously, we have shown that TLR4 is expressed in the taste epithelium [16]. It is conceivable that LPS may directly or indirectly activate cells in taste epithelium and stimulate the production of inflammatory cytokines, which may inhibit taste progenitor cell proliferation. Future studies will identify the molecular pathways responsible for this inhibition.

PCR array experiments showed that LPS downregulated the expression of Brca1 and Chek1 in the taste epithelium; both genes are involved in the detection and repair of DNA damages [60]. Reduced activities of Brca1 and Chek1 may loosen the control of cell cycle checkpoints for DNA defects and result in an accumulation of DNA mutations in proliferating cells. In addition, it is known that inflammation, through the generation of reactive oxygen species, increases DNA damage [67]. These effects together may impose a threat to genome integrity and increase susceptibility to tumorigenesis and accumulation of somatic mutations in taste tissues [8,67].

### Inflammation and taste cell degeneration and turnover

P53, Bax, and Caspase-2 have been implicated in the physiological turnover of taste bud cells [28,29]. The taste buds from Bax-deficient mice contain more than twice the normal number of taste cells [29]. Other apoptosis-related genes, such as caspase-3, 6, 7, 8, and 9, are also detected in the taste buds and some show higher levels of expression in taste cells than in nontaste cells [17]. We previously demonstrated that IFN-α and γ stimulate the activation of caspase-3 and increase apoptosis in taste buds [15]. In this study, we showed that LPS injection rapidly induced the expression of IFN-γ in TrpM5-positive taste receptor cells (Figure 1, 2), suggesting that LPS may accelerate cell death of some taste bud cells through the IFN pathways.

Indeed, BrdU pulse-chase experiments revealed that LPS-induced inflammation moderately shortened the average life span of taste bud cells (Figure 4A), indicating that cell death occurs faster in taste buds after LPS treatment. This effect of LPS seems modest. However, it is possible that LPS administration shortens the life span of only a subset of taste cells. Therefore, although the average life span of all BrdU-labeled taste cells was not markedly altered, some types of taste cells might have been affected to a greater level than the average. The restricted induction of IFN-γ in a subset of taste cells is in line with this notion (Figure 2). Previous research showed that different subtypes of taste cells may have different cell turnover rates [18,26,27]. Light or type II taste receptor cells, which express the sweet, bitter, and umami receptors and their downstream signaling components (e.g., TrpM5), seem to have longer life spans than do dark or type I cells [18,26]. It remains to be determined whether LPS-induced inflammation selectively accelerates the turnover rates of only subtypes of taste bud cells. Furthermore, mechanisms other than increased cell death may also contribute to the shortened average life span of taste cells. For instance, the population of different types of taste cells may be altered by LPS towards one with a shorter life span in the buds.

It should be noted that the acute inflammation induced by LPS also activates anti-inflammatory pathways following the peak of inflammatory response. These anti-inflammatory mechanisms dampen immune reactions and facilitate resolution of inflammation. In taste tissues, the presence of such pathways may reduce the effects of inflammation and promote the recovery of taste bud structure and function after acute infections and other inflammatory insults. Effects of LPS on taste cells are likely to be the consequences of the orchestrated action of pro- and anti-inflammatory pathways.

### Inflammation and taste disorders

Compared to nontaste lingual epithelial cells, taste bud cells are enriched with a number of molecules that play key roles in the process of inflammation. Several TLRs, including the LPS receptor TLR4, are preferentially expressed in the taste buds [16]. These higher levels of expression of TLRs may account for the more robust production of inflammatory cytokines in the taste epithelium after an inflammatory challenge. Preferential expression in the taste buds was also observed for some chemokines, such as CXCL14 and IL-8, as well as cytokine receptors [15,17]. This expression pattern may normally protect taste cells from pathogens, but can also lead to detrimental consequences under excessive or chronic inflammatory conditions.

As shown in this study, LPS-induced inflammation, which mimics Gram-negative bacterial infections and releases a plethora of inflammatory cytokines [50,68], has an adverse impact on taste bud structure. LPS treatment has been shown to alter the preference for sweet and bitter taste compounds [69]. Although whether these behavioral changes are related to the changes in taste buds has not been determined, the present study suggests that inflammation, an underlying condition in various diseases associated with taste disorders, affects taste bud cells and may contribute to the development of taste dysfunction.

## Conclusions

To our knowledge, this is the first study to investigate the effects of inflammation on taste bud cell turnover. LPS-induced systemic inflammation strongly inhibits taste progenitor cell proliferation and results in reduced number of newborn cells entering the taste buds. This inhibition correlates with decreased expression of Ki67, cyclin B2, and E2F1 in circumvallate and foliate epithelia; the latter two genes play important roles in cell proliferation. In addition, LPS modestly shortens the average turnover period of taste bud cells. These results support the hypothesis that inflammation contributes to the development of taste disorders associated with infections, autoimmune diseases, and cancer.

## Methods

### Animals

C57BL/6 mice were purchased from Jackson Laboratory (Bar Harbor, ME) and used for most of the experiments except immunostaining using TrpM5-GFP mice. The generation and characterization of TrpM5-GFP mice were reported previously [52,53]. Mice were housed in a climate-controlled environment at the animal care facility of the Monell Chemical Senses Center. Studies were performed according to protocols approved by the Monell Chemical Senses Center Institutional Animal Care and Use Committee.

### Reagents

The anti-BrdU monoclonal antibody (G3G4) developed by S. J. Kaufman was obtained from the Developmental Studies Hybridoma Bank developed under the auspices of the National Institute of Child Health and Human Development and maintained by The University of Iowa Department of Biological Sciences (Iowa City, IA) [70,71]. Rabbit and goat polyclonal antibodies against KCNQ1 were purchased from Millipore (Billerica, MA) (AB5932) and Santa Cruz Biotechology (Santa Cruz, CA) [54,72]. Mouse monoclonal antibodies against Ki67 and K14 were purchased from BD Biosciences (San Jose, CA) (clone B56) and Millipore, respectively [47,73]. Affinity-purified rabbit polyclonal antibody against IFN-γ, as well as control rabbit and goat IgG, were purchased from PeproTech (Rocky Hill, NJ). Affinity-purified goat polyclonal antibody against TNF-α was purchased from R&D Systems (Minneapolis, MN) [74]. Cyanine 3 (Cy3)-conjugated goat anti-rabbit antibody, Cy5-conjugated goat anti-mouse antibody, and Cy5-conjugated donkey anti-goat antibody were purchased from Jackson ImmunoResearch (West Grove, PA). Mouse Alexa 488 Zenon Antibody Labeling Kit and Alexa 488-conjugated donkey anti-goat antibody were purchased from Invitrogen (Carlsbad, CA). BrdU and LPS were purchased from Sigma (St. Louis, MO).

### Quantitative real-time RT-PCR analysis

Six and 24 hours after intraperitoneal injection of PBS (Invitrogen, catalog no. #14040-117) or LPS (5 mg/kg in PBS), mice were sacrificed and tongue epithelium was prepared as previously described [15]. Total RNA was extracted using Absolutely RNA Microprep Kit (Stratagene, Cedar Creek, TX) from peeled-off epithelial pieces that either lacked taste buds (excised from within the intermolar eminence) or contained foliate or circumvallate taste buds. Epithelial pieces containing foliate or circumvallate taste buds from 4-5 mice in each group were pooled together as the taste epithelium sample. Approximately equal amounts of total RNA from these samples were reverse transcribed into cDNA using Superscript III reverse transcriptase (Invitrogen). Quantitative real-time PCR was set up using Power SYBR Green PCR Master Mix (Applied Biosystems) and run on an ABI PRISM 7000 Sequence Detection System (Applied Biosystems). Relative quantification of gene expression was performed using ABI software, which was based on the 2^-ΔΔCt ^method [75]. β-actin was used as the endogenous control gene for these analyses. RT-PCR primers were designed to place the forward primer and the reverse primer in separate exons of each gene (see Table 1). The resulting DNA products were run on agarose gels to confirm the size of the DNA products. These experiments were repeated three times.

### BrdU pulse-chase experiment for taste bud cell turnover

The experiment was carried out with male C57BL/6 mice about 6 weeks of age when the experiment began. Five doses of BrdU (20 mg/kg per dose) were given to each mouse by intraperitoneal injections over a 12 h period (interinjection intervals were 3 h). Half of these mice also received a single injection of LPS (5 mg/kg dissolved in PBS) 1 h after the first BrdU injection, and the other half received an injection of vehicle (PBS buffer) as control. Mice were sacrificed at 1, 2, 3, 4, 5, 6, 7, 8, 10, 12, 14, 16, 18, 20, 25, and 30 days after the first BrdU injection. Five mice per group were sacrificed at each time for days 1-3, and 2 mice per group for days 4-30. Tongues were fixed in 4% paraformaldehyde (PFA)/PBS solution for 1 h on ice and transferred to 20% sucrose/PBS solution for an overnight incubation at 4°C. Tissues were then mounted and cryosectioned into 10 μm serial sections of circumvallate papillae.

Representative circumvallate sections (6-8 sections) were selected from each mouse for immunostaining. The positions of these sections (from dorsal to ventral position on the tongue) were approximately the same for each mouse across all time points and groups. Sections were washed twice in PBS solution containing 0.3% Triton X-100, followed by rinses with deionized H_2_O and then incubation in 4 N HCl for 20 min. After a second round of washes with PBS containing 0.3% Triton X-100 (pH 7.4), the sections were incubated with a blocking buffer (3% bovine serum albumin, 0.3% Triton X-100, 2% goat serum, and 0.1% sodium azide in PBS) at 4°C overnight. The anti-BrdU mouse monoclonal antibody was labeled with the Alexa 488 Zenon Mouse Antibody Labeling Kit (Invitrogen) following the protocol recommended by the manufacturer. The freshly labeled antibody was added to the slides within 30 min of preparation and incubated at room temperature for 2 h. The sections were washed twice with PBS containing 0.3% Triton X-100 solution and once with PBS (pH 7.4) and then postfixed in freshly prepared 4% PFA/PBS for 15 min at room temperature. The slides were washed three more times and blocked with the blocking buffer at room temperature for 1 h, and then incubated with rabbit anti-KCNQ1 antibody at 4°C overnight. Cy3-conjugated goat anti-rabbit secondary antibody (Jackson ImmunoResearch) was added to sections for 40 min. Sections were washed again and mounted with Vectashield H-1000 mounting medium (Vector Laboratories, Burlingame, CA) and imaged with a Leica confocal microscope.

BrdU-labeled cells in the circumvallate epithelium were classified as either perigemmal or intragemmal cells, with the taste bud profiles defined by the KCNQ1 immunostaining. BrdU-labeled cells had to be fully surrounded by KCNQ1 staining in order to be counted as intragemmal cells. The number of BrdU-labeled intragemmal cells per taste bud profile for each group at each time point was averaged and plotted as a time course (see Figure 4A), which was used to estimate the taste bud cell entry time and the turnover period. The average taste bud cell entry time was determined as the time from the first BrdU injection to the point when 50% of the peak number of labeled cells entered the taste buds. The taste bud cell turnover period was calculated as the time from the first half-maximum time point on the ascending slope to the second half-maximum time point on the descending slope of the time course. For days 1-3, additional circumvallate sections were processed and the BrdU-labeled cells were counted. The numbers from these sections were averaged with the ones described above and the results are summarized in Figure 4B.

For counting BrdU-labeled cells in the perigemmal regions of circumvallate epithelium, we selected two 150 μm × 70 μm regions on each circumvallate section, one from each side of the vallate trench. The average numbers of BrdU-labeled perigemmal cells/mm^2 ^were calculated and plotted against time (see Figure 5A). For day 1, additional sections from each group were counted and the average numbers are shown in Figure 5B. The average cell turnover period was calculated from the half-maximum ascending and descending time points, as described above for taste bud cells. For all images, the brightness of BrdU staining was adjusted to similar levels based on the intensity count by the imaging software. The same person performed all the counts in order to maintain a consistent standard for counting.

### Immunostaining of TNF-α and IFN-γ

Six hours after LPS (5 mg/kg) injection, mice were sacrificed and tongues were removed and fixed in 4% PFA/PBS solution. Tissues were then processed for cryosectioning. Circumvallate sections were washed three times with PBS containing 0.3% Triton X-100 and then incubated with a permeabilization buffer (0.1% saponin and 0.009% sodium azide) at room temperature for 1 h, followed by an incubation with a blocking buffer (3% bovine serum albumin, 0.3% Triton X-100, 2% goat or horse serum, and 0.1% sodium azide in PBS) containing 0.1% saponin at room temperature for 1 h. The sections were then incubated with either an affinity-purified rabbit antibody against IFN-γ or an affinity-purified goat antibody against TNF-α in blocking buffer at room temperature for 1 h or at 4°C overnight. The sections were washed and further incubated with a Cy3-conjugated goat anti-rabbit secondary antibody (for IFN-γ staining), an Alexa 488-conjugated donkey anti-goat antibody (for TNF-α staining), or a Cy5-conjugated donkey anti-goat antibody (for colocalization of TNF-α with TrpM5-GFP) at room temperature for 1 h. Sections were washed and mounted with Vectashield. Images were taken using a Leica confocal microscope. For control experiments, we used non-specific normal rabbit or goat IgG to replace the antibodies against IFN-γ or TNF-α and followed by incubations with the same secondary antibodies mentioned above. For antigen blocking experiments, antibodies against IFN-γ or TNF-α were pre-incubated with purified recombinant murine IFN-γ or TNF-α (PeproTech), respectively. These mixtures were then added to the tissue sections and processed as described above.

### Ki67 immunostaining and cell counting

Twenty-four hours after PBS or LPS (5 mg/kg in PBS) injection, C57BL/6 mice were sacrificed and tongues were removed and frozen in mounting medium. Frozen sections (10-μm) were prepared and fixed in cold acetone for 30 sec. Sections were air dried and washed three times with PBS containing 0.3% Triton X-100. After incubation at room temperature for 2 h with blocking buffer, the sections were further incubated with a rabbit polyclonal anti-KCNQ1 antibody at 4°C overnight. After washing with PBS/0.3% Triton X-100 solution, a Cy3-conjugated goat anti-rabbit secondary antibody was added to the sections and incubated for 60 min. Mouse monoclonal anti-Ki67 antibody was labeled with Alexa 488 Zenon Mouse IgG Labeling Kit following the manufacturer's protocol. The labeled antibody was added to the sections and incubated at room temperature for 2 h. Sections were washed and mounted with Vectashield. Images were taken immediately using a Leica confocal microscope. Ki67-labeled cells immediately surrounding a taste bud (defined by the KCNQ1 staining) in the circumvallate epithelium were counted. The average numbers of Ki67-labeled cells per taste bud profile were calculated for both PBS and LPS-treated mice.

### Gene expression analysis using PCR array

Twenty-four hours after intraperitoneal injection of PBS or LPS, mice were sacrificed and tongue epithelium was peeled off as previously described [15]. Total RNA was extracted from nontaste lingual epithelium or epithelium containing foliate or circumvallate taste buds. Approximately equal amounts of total RNA from these samples were reverse transcribed into cDNA using Superscript III reverse transcriptase (Invitrogen). Quantitative real-time PCR was set up using Power SYBR Green PCR Master Mix (Applied Biosystems) and PCR primer sets from the Mouse Cell Cycle RT^2 ^Profiler PCR Array (PAMM-020A, SABiosciences). PCR was performed on an ABI PRISM 7000 Sequence Detection System (Applied Biosystems). The results were analyzed using the SABiosciences PCR Array Data Analysis Web Portal. The scatter plot in Figure 7A was also generated using these analysis tools.

## Authors' contributions

ZJC performed BrdU immunostaining, data acquisition, and data analysis. AK carried out qPCR studies, immunostaining, and data analysis. LH and JB were involved in experimental design and manuscript preparation. LH also participated in data analysis. HW performed some qPCR analysis, immunostaining, data processing and experimental design. ZJC and HW drafted the manuscript. All authors read and approved the manuscript.

## Supplementary Material

Additional File 1 **Agarose gel image of qPCR products**. qPCR products for TNF-α, IFN-γ, IL-6, IL-12, MCP-1, and IL-1β were analyzed by agarose gel electrophoresis. Duplicate samples for LPS-treated circumvallate and foliate (CV+F) epithelia were included. The 1 Kb Plus DNA Ladder (Invitrogen) serves as reference for DNA size (M). qPCR primers and the sizes of the predicted PCR products are listed in Table 1. Click here for file

Additional File 2 **Colocalization of Ki67- and K14-positive cells**. Confocal fluorescent images of circumvallate papillae stained with antibodies against Ki67 (green) and K14 (red). Higher magnification images are shown in bottom panels. Ki67-positive cells in circumvallate epithelium are also positive for K14 (indicated by arrows). Scale bars, 25 μm. Click here for file

Additional File 3 **Mouse cell cycle PCR array data**. This table lists all the PCR primer sets in the array, including primers for 84 genes involved in cell cycle regulation (positions A01-G12), 5 genes used as endogenous controls for quantification of gene expression (positions H01-H05), and 7 sets used for experimental quality control (position H06-H12). Relative expression (in fold, LPS vs. PBS) is shown in the last column: positive numbers indicate increased expression, and negative numbers decreased expression, in the circumvallate epithelium of LPS-treated mice compared with PBS-treated mice. Click here for file
