# Supplementary figures and images for: Lipopolysaccharide-induced inflammation attenuates taste progenitor cell proliferation and shortens the life span of taste bud cells
Source: BMC Neurosci. 2010 Jun 10;11:72. doi: 10.1186/1471-2202-11-72 (PMC2898829; doi:10.1186/1471-2202-11-72)

Additional File 1

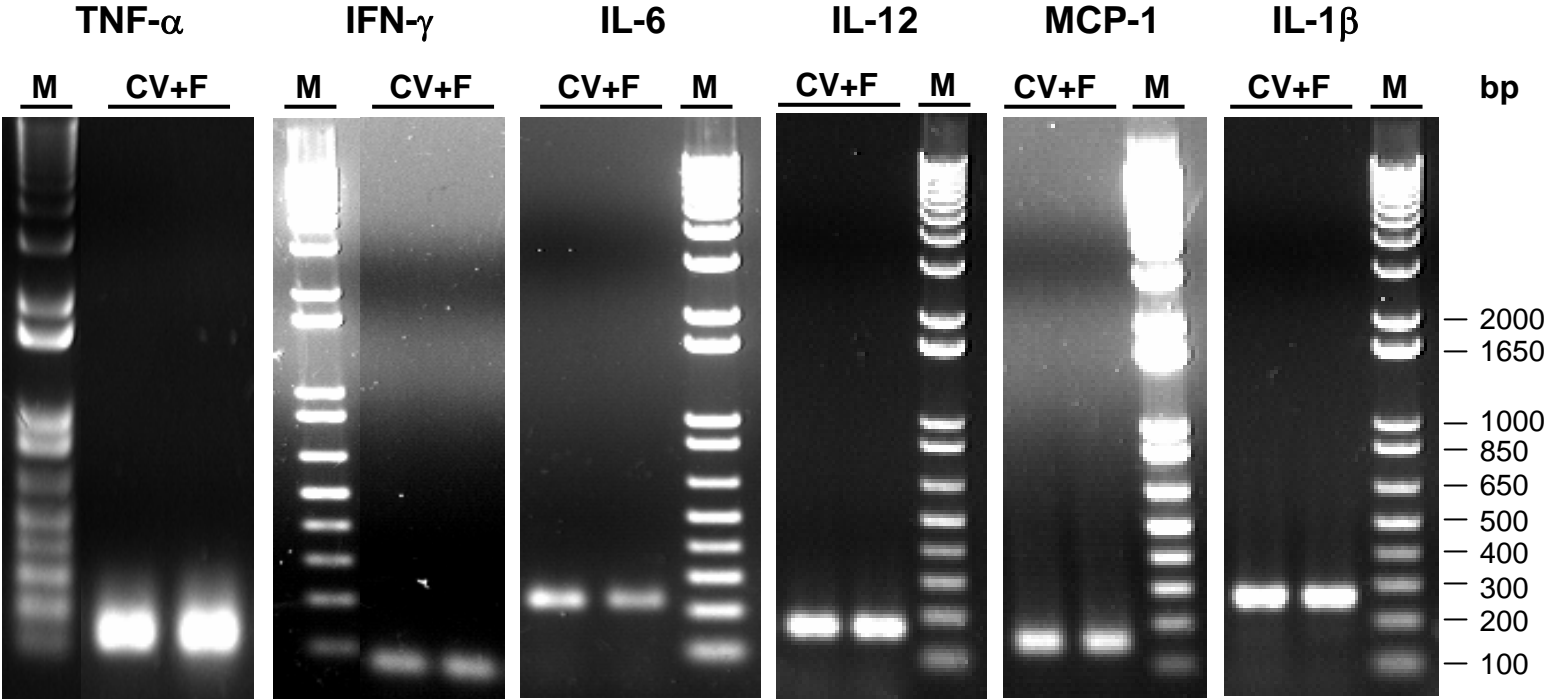

Supplement: Additional File 1 — Agarose gel image of qPCR products. qPCR products for TNF-α, IFN-γ, IL-6, IL-12, MCP-1, and IL-1β were analyzed by agarose gel electrophoresis. Duplicate samples for LPS-treated circumvallate and foliate (CV+F) epithelia were included. The 1 Kb Plus DNA Ladder (Invitrogen) serves as reference for DNA size (M). qPCR primers and the sizes of the predicted PCR products are listed in Table 1. [file 1471-2202-11-72-S1.PDF]

## Additional File 2

**Ki67**

**K14**

**Merge**

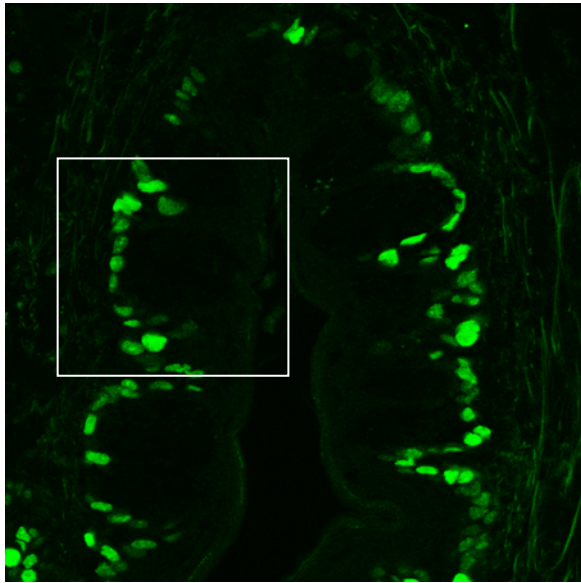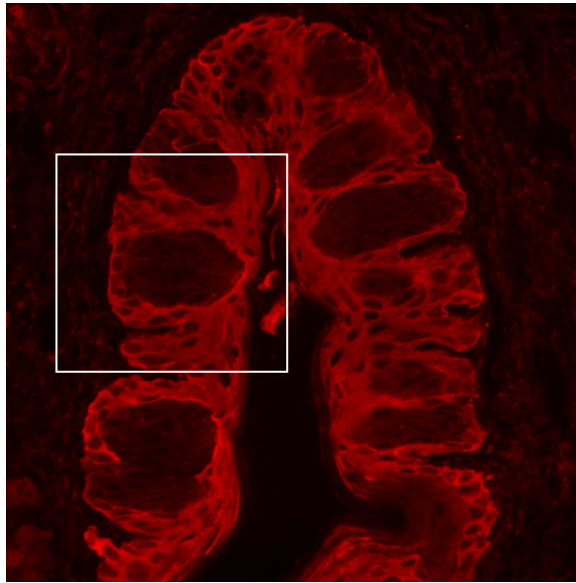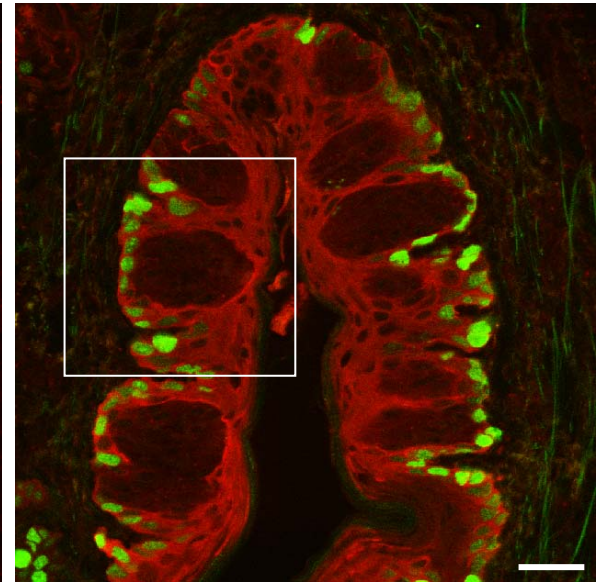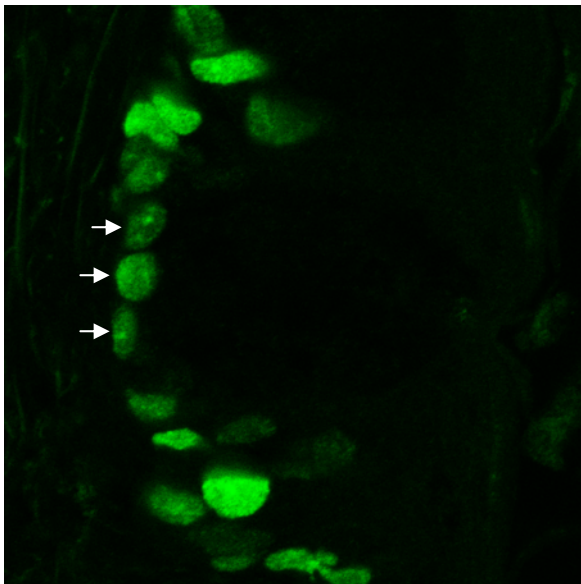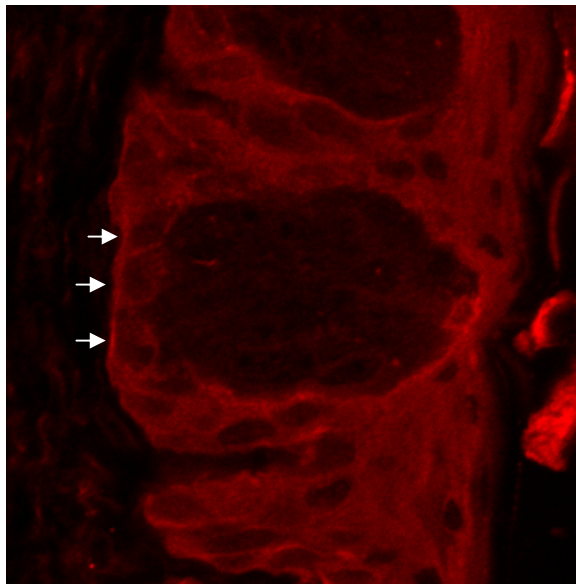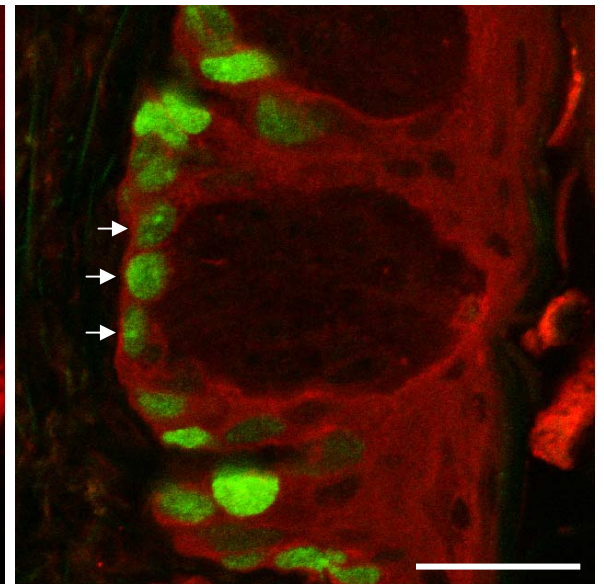

Supplement: Additional File 2 — Colocalization of Ki67- and K14-positive cells. Confocal fluorescent images of circumvallate papillae stained with antibodies against Ki67 (green) and K14 (red). Higher magnification images are shown in bottom panels. Ki67-positive cells in circumvallate epithelium are also positive for K14 (indicated by arrows). Scale bars, 25 μm. [file 1471-2202-11-72-S2.PDF]
