# Supplementary material for: Lipopolysaccharide-induced inflammation attenuates taste progenitor cell proliferation and shortens the life span of taste bud cells
Source: BMC Neurosci. 2010 Jun 10;11:72. doi: 10.1186/1471-2202-11-72 (PMC2898829; doi:10.1186/1471-2202-11-72)
Supplement: Additional File 3 — Mouse cell cycle PCR array data. This table lists all the PCR primer sets in the array, including primers for 84 genes involved in cell cycle regulation (positions A01-G12), 5 genes used as endogenous controls for quantification of gene expression (positions H01-H05), and 7 sets used for experimental quality control (position H06-H12). Relative expression (in fold, LPS vs. PBS) is shown in the last column: positive numbers indicate increased expression, and negative numbers decreased expression, in the circumvallate epithelium of LPS-treated mice compared with PBS-treated mice. [file 1471-2202-11-72-S3.PDF]

## Additional File 1. Mouse cell cycle PCR array data:

| Position | Gene Symbol | GenBank | Gene Description | Expression (fold) |
|---|---|---|---|---|
| A01 | Abl1 | NM_009594 | C-abl oncogene 1, receptor tyrosine kinase | -3.3846 |
| A02 | Ak1 | NM_021515 | Adenylate kinase 1 | 1.6575 |
| A03 | Apbb1 | NM_009685 | Amyloid beta (A4) precursor protein-binding, family B, member 1 | -1.0644 |
| A04 | Atm | NM_007499 | Ataxia telangiectasia mutated homolog (human) | 1.9766 |
| A05 | Brca1 | NM_009764 | Breast cancer 1 | -4.4753 |
| A06 | Brca2 | NM_009765 | Breast cancer 2 | -2.3702 |
| A07 | Camk2a | NM_177407 | Calcium/calmodulin-dependent protein kinase II alpha | 2.8011 |
| A08 | Camk2b | NM_007595 | Calcium/calmodulin-dependent protein kinase II, beta | 1.6632 |
| A09 | Casp3 | NM_009810 | Caspase 3 | -1.3004 |
| A10 | Ccna1 | NM_007628 | Cyclin A1 | -1.9119 |
| A11 | Ccna2 | NM_009828 | Cyclin A2 | 1.5052 |
| A12 | Ccnb1 | NM_172301 | Cyclin B1 | 1.5052 |
| B01 | Ccnb2 | NM_007630 | Cyclin B2 | -8.7787 |
| B02 | Ccnc | NM_016746 | Cyclin C | -1.6876 |
| B03 | Ccnd1 | NM_007631 | Cyclin D1 | -1.4459 |
| B04 | Ccne1 | NM_007633 | Cyclin E1 | -1.2492 |
| B05 | Ccnf | NM_007634 | Cyclin F | -2.2689 |
| B06 | Cdc25a | NM_007658 | Cell division cycle 25 homolog A (S. pombe) | -1.1487 |
| B07 | Cdk2 | NM_016756 | Cyclin-dependent kinase 2 | 1.041 |
| B08 | Cdk4 | NM_009870 | Cyclin-dependent kinase 4 | -1.425 |
| B09 | Cdk5rap1 | NM_025876 | CDK5 regulatory subunit associated protein 1 | 1.0338 |
| B10 | Cdkn1a | NM_007669 | Cyclin-dependent kinase inhibitor 1A (P21) | -1.0695 |
| B11 | Cdkn1b | NM_009875 | Cyclin-dependent kinase inhibitor 1B | 1.5052 |
| B12 | Cdkn2a | NM_009877 | Cyclin-dependent kinase inhibitor 2A | 3.6276 |
| C01 | Chek1 | NM_007691 | Checkpoint kinase 1 homolog (S. pombe) | -6.8781 |
| C02 | Cks1b | NM_016904 | CDC28 protein kinase 1b | -1.426 |
| C03 | Ddit3 | NM_007837 | DNA-damage inducible transcript 3 | 1.305 |
| C04 | Dnajc2 | NM_009584 | DnaJ (Hsp40) homolog, subfamily C, member 2 | 1.4948 |
| C05 | Dst | NM_134448 | Dystonin | -1.432 |
| C06 | E2f1 | NM_007891 | E2F transcription factor 1 | -4.2516 |
| C07 | E2f2 | NM_177733 | E2F transcription factor 2 | -1.8239 |
| C08 | E2f3 | NM_010093 | E2F transcription factor 3 | -1.3938 |

| Position | Gene Symbol | GenBank | Gene Description | Expression (fold) |
|---|---|---|---|---|
| C09 | E2f4 | NM_148952 | E2F transcription factor 4 | 1.0014 |
| C10 | Gadd45a | NM_007836 | Growth arrest and DNA-damage-inducible 45 alpha | 1.2535 |
| C11 | Gpr132 | NM_019925 | G protein-coupled receptor 132 | -1.5273 |
| C12 | Hus1 | NM_008316 | Hus1 homolog (S. pombe) | 1.0338 |
| D01 | Inha | NM_010564 | Inhibin alpha | 2.4915 |
| D02 | Itgb1 | NM_010578 | Integrin beta 1 (fibronectin receptor beta) | -1.7267 |
| D03 | Macf1 | XM_884922 | Microtubule-actin crosslinking factor 1 | -1.5115 |
| D04 | Mad2l1 | NM_019499 | MAD2 mitotic arrest deficient-like 1 (yeast) | -2.1243 |
| D05 | Mcm2 | NM_008564 | Minichromosome maintenance deficient 2 mitotin (S. cerevisiae) | -1.7642 |
| D06 | Mcm3 | NM_008563 | Minichromosome maintenance deficient 3 (S. cerevisiae) | -2.558 |
| D07 | Mcm4 | NM_008565 | Minichromosome maintenance deficient 4 homolog (S. cerevisiae) | -2.3311 |
| D08 | Mdm2 | NM_010786 | Transformed mouse 3T3 cell double minute 2 | 1.0882 |
| D09 | Mki67 | XM_001000692 | Antigen identified by monoclonal antibody Ki 67 | -7.2 |
| D10 | Mre11a | NM_018736 | Meiotic recombination 11 homolog A (S. cerevisiae) | -1.108 |
| D11 | Msh2 | NM_008628 | MutS homolog 2 (E. coli) | -1.1559 |
| D12 | Mtbp | NM_134092 | Mdm2, transformed 3T3 cell double minute p53 binding protein | -2.4217 |
| E01 | Myb | NM_010848 | Myeloblastosis oncogene | -1.5627 |
| E02 | Nek2 | NM_010892 | NIMA (never in mitosis gene a)-related expressed kinase 2 | -1.6313 |
| E03 | Nfatc1 | NM_016791 | Nuclear factor of activated T-cells, cytoplasmic, calcineurin-dependent 1 | 1.2431 |
| E04 | Notch2 | NM_010928 | Notch gene homolog 2 (Drosophila) | -1.0077 |
| E05 | Npm2 | NM_181345 | Nucleophosmin/nucleoplasmin 2 | -1.3278 |
| E06 | Pcna | NM_011045 | Proliferating cell nuclear antigen | -1.7041 |
| E07 | Pes1 | NM_022889 | Pescadillo homolog 1, containing BRCT domain (zebrafish) | 1.1431 |
| E08 | Pkd1 | NM_013630 | Polycystic kidney disease 1 homolog | 1.0636 |
| E09 | Pmp22 | NM_008885 | Peripheral myelin protein 22 | -1.244 |
| E10 | Ppm1d | NM_016910 | Protein phosphatase 1D magnesium-dependent, delta isoform | 1.0497 |
| E11 | Ppp2r3a | XM_001471965 | Protein phosphatase 2 (formerly 2A), regulatory subunit B'', alpha | -1.8063 |
| E12 | Ppp3ca | NM_008913 | Protein phosphatase 3, catalytic subunit, alpha isoform | 1.0331 |
| F01 | Prm1 | NM_013637 | Protamine 1 | 1.5713 |
| F02 | Rad17 | NM_011233 | RAD17 homolog (S. pombe) | -1.0688 |
| F03 | Rad21 | NM_009009 | RAD21 homolog (S. pombe) | -1.1672 |
| F04 | Rad51 | NM_011234 | RAD51 homolog (S. cerevisiae) | -3.2378 |
| F05 | Rad9 | NM_011237 | RAD9 homolog (S. pombe) | -1.4641 |

| Position | Gene Symbol | GenBank | Gene Description | Expression (fold) |
|---|---|---|---|---|
| F06 | Ran | NM_009391 | RAN, member RAS oncogene family | -1.0182 |
| F07 | Rbl1 | NM_011249 | Retinoblastoma-like 1 (p107) | -1.8947 |
| F08 | Rbl2 | NM_011250 | Retinoblastoma-like 2 | -1.2388 |
| F09 | Sesn2 | NM_144907 | Sestrin 2 | -1.1447 |
| F10 | Sfn | NM_018754 | Stratifin | -1.3204 |
| F11 | Shc1 | NM_011368 | Src homology 2 domain-containing transforming protein C1 | 1.0042 |
| F12 | Skp2 | NM_013787 | S-phase kinase-associated protein 2 (p45) | -2.2069 |
| G01 | Slfn1 | NM_011407 | Schlafen 1 | 1.0119 |
| G02 | Smc1a | NM_019710 | Structural maintenance of chromosomes 1A | -1.3813 |
| G03 | Stag1 | NM_009282 | Stromal antigen 1 | 1.1479 |
| G04 | Sumo1 | NM_009460 | SMT3 suppressor of mif two 3 homolog 1 (yeast) | -1.1696 |
| G05 | Taf10 | NM_020024 | TAF10 RNA polymerase II, TATA box binding protein (TBP)-associated factor | -1.172 |
| G06 | Terf1 | NM_009352 | Telomeric repeat binding factor 1 | 1.2125 |
| G07 | Tfdp1 | NM_009361 | Transcription factor Dp 1 | -1.5529 |
| G08 | Psmg2 | NM_134138 | Proteasome (prosome, macropain) assembly chaperone 2 | -1.2702 |
| G09 | Trp53 | NM_011640 | Transformation related protein 53 | 1.1527 |
| G10 | Trp63 | NM_011641 | Transformation related protein 63 | 1.0154 |
| G11 | Tsg101 | NM_021884 | Tumor susceptibility gene 101 | 1.1818 |
| G12 | Wee1 | NM_009516 | WEE 1 homolog 1 (S. pombe) | -1.59 |
| H01 | Gusb | NM_010368 | Glucuronidase, beta | 1.5032 |
| H02 | Hprt1 | NM_013556 | Hypoxanthine guanine phosphoribosyl transferase 1 | 1.1599 |
| H03 | Hsp90ab1 | NM_008302 | Heat shock protein 90 alpha (cytosolic), class B member 1 | -1.2167 |
| H04 | Gapdh | NM_008084 | Glyceraldehyde-3-phosphate dehydrogenase | -1.4035 |
| H05 | Actb | NM_007393 | Actin, beta | -1.021 |
| H06 | MGDC | SA_00106 | Mouse Genomic DNA Contamination | |
| H07 | RTC | SA_00104 | Reverse Transcription Control | |
| H08 | RTC | SA_00104 | Reverse Transcription Control | |
| H09 | RTC | SA_00104 | Reverse Transcription Control | |
| H10 | PPC | SA_00103 | Positive PCR Control | |
| H11 | PPC | SA_00103 | Positive PCR Control | |
| H12 | PPC | SA_00103 | Positive PCR Control | |
